# Feeding habits and malaria parasite infection of *Anopheles* mosquitoes in selected agroecological areas of Northwestern Ethiopia

**DOI:** 10.1186/s13071-024-06496-y

**Published:** 2024-10-03

**Authors:** Aklilu K. Belay, Abebe Asale, Catherine L. Sole, Abdullahi A. Yusuf, Baldwyn Torto, Clifford M. Mutero, David P. Tchouassi

**Affiliations:** 1 International Centre of Insect Physiology and Ecology, P.O. Box 30772-00100, Nairobi, Kenya; 2 International Centre of Insect Physiology and Ecology, P.O. Box 30772-5689, Addis Ababa, Ethiopia; 3 Department of Zoology and Entomology, University of Pretoria, Private Bag X0028, Pretoria, South Africa; 4 School of Health Systems and Public Health, University of Pretoria, Private Bag X0028, Pretoria, South Africa

**Keywords:** Highland ecology, Primary and secondary malaria vectors, Human blood index, Bovine blood index, *Anopheles arabiensis*, Vector behavior

## Abstract

**Background:**

Surveillance of the host–anopheline mosquitoes’ interaction is important for assessing malaria transmission risk and guiding vector control. We assume that changes in malaria vector species’ feeding habits, as well as the surrounding environment, have a substantial impact on varied malaria transmission. In this study, we determined the vertebrate host feeding patterns of anopheline mosquitoes to characterize entomologic risk factors for malaria in Jabi Tehnan, Northwestern Ethiopia.

**Methods:**

Blood-fed anophelines surveyed during malaria surveillance in Jabi Tehnan district of northwestern Ethiopia were utilized in this study. They were collected using Centers for Disease Control and Prevention (CDC) light traps deployed in selected households per village, placed indoors and outdoors, spanning three agroecological settings (dry mountain, plateau, and semiarid highlands) between June 2020 and May 2021. The engorged mosquitoes were analyzed for host blood meal sources and *Plasmodium* infection via polymerase chain reaction (PCR) and/or sequencing. Infection rates and bovine and human blood indices were calculated and compared for abundant species; between indoors and outdoors and between agroecology using a chi-squared test for equality of proportion in R package at a significant level of *p* ≤ 0.05.

**Results:**

A total of 246 mosquitoes were successfully typed (indoor, 121; outdoor, 125), with greater relative abundance indoors in mountain and plateau highlands, and outdoors in semiarid areas. Despite ecological differences in blood-fed capture rates, cattle served as the most utilized blood meal source by 11 anopheline species with an overall bovine blood index (BBI) of 74.4%. This trend was dictated by *Anopheles gambiae* s.l. (198/246; BBI = 73.7%), which exhibited the most plastic feeding habits that included humans (human blood index = 15.7%) and other livestock and rodents. A total of five anopheline species (*An. gambiae* s.l., *An. funestus* s.l., *An. coustani* s.l., *An. pretoriensis*, and *An. pharoensis*) fed on humans, of which the first three were found infected with *Plasmodium* parasites. Most of the infected specimens were *An. arabiensis* (5.6%, 11/198) and had recently fed mainly on cattle (72.7%, 8/11); one each of infected *An. funestus* s.l. and *An. coustani* s.l. had fed on humans and cattle, respectively.

**Conclusions:**

The results demonstrate communal feeding on cattle by anophelines including primary and secondary malaria vectors. This study also indicates the importance of cattle-targeted interventions for sustainable control of malaria vectors in the study areas.

**Graphical abstract:**

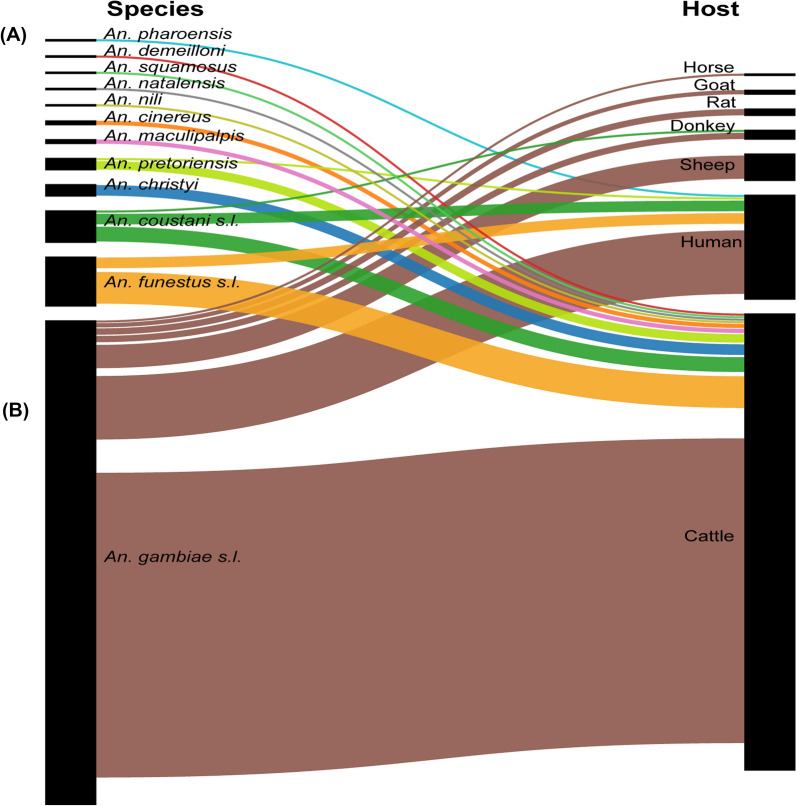

## Background

Malaria remains a significant public health issue in sub-Saharan Africa, with transmission patterns varying across ecological landscapes [1]. The main vectors, *Anopheles gambiae* s.l. and *An. funestus* s.l., drive most of the transmission due to high vectorial capacity [2, 3]. Female anophelines seek vertebrate hosts to obtain blood for their reproductive needs. This biological process, however, serves as the precursor to pathogen infection and transmission. More importantly, this trait has evolved in certain mosquito species to highly utilize humans as the primary source of blood meal, making them deadly vectors of human diseases [4, 5]. Well-known examples include the mosquitoes *An. gambiae* s.l. and *Aedes aegypti,* the efficient vectors of malaria and dengue, respectively [4, 6, 7]. Despite this apparent inherent effect, patterns of blood-feeding can be highly variable in different ecological settings and are often modulated by factors such as host availability (abundance and diversity) and vector behavior [4, 6, 7].

There are indications that host availability affects vector fitness and longevity and could shape vector distribution and risk of vector-borne diseases [8, 9]. Thus, analysis of blood-feeding patterns is important when assessing the ecological spread and sustenance of vector-borne diseases such as malaria and in revealing insights into vectorial diseases [8, 9]. Studies show that habits of blood-feeding and resting behavior of anopheline species can vary with their relative importance in malaria transmission [10, 11]. Highly opportunistic feeding, mainly on cattle, has been reported for *An. arabiensis*, the primary vector of malaria in most parts of Ethiopia [10, 12, 13] and parts of Kenya, Ghana, and Tanzania [14–16]. The newly introduced anopheline species in Africa, *An. stephensi*, exhibits strong zoophagic tendencies reflected in its low infectivity to human *Plasmodium* parasites [17]. *Anopheles* mosquitoes feed on a wide variety of hosts, including humans, cattle, other domestic and wild animals, rodents, and birds [8, 10, 18, 19].

To date, the network of host utilization remains poorly described for most primary and secondary malaria vectors. Factors such as urbanization, limited intervention, variations in agroecology, and housing standards are likely to alter the risk of human exposure to mosquito bites and malaria [20–22]. Studies conducted in Tanzania found that the risk of outdoor malaria exposure is higher for older/school-age children and adults who are highly engaged in outdoor activities [23]. Investigating the blood-feeding patterns of malaria vectors across diverse ecological niches is vital in planning interventions [4, 24, 25]. In addition, estimates of human blood index (HBI) and bovine blood index (BBI) and human outdoor and indoor feeding can be dependent on mosquito resting habits and environmental conditions [24]. Addressing these research gaps can aid in advancing our understanding of malaria transmission dynamics and informing the development of more effective control measures adjusted to specific ecological contexts.

The focus of this study was to examine the blood-feeding habits of the local *Anopheles* species, encountered during a baseline survey to improve understanding of entomologic risk factors of malaria in the Jabi Tehnan district, situated in Northwestern Ethiopia. The present study analyzed the engorged cohort only of the anopheline mosquitoes sampled and reported in a recent article [26]. We hypothesize that the variability in malaria transmission within the district is significantly influenced by differences in the feeding habits of malaria vector species and shaped by the distinct agroecological zones. Blood-feeding patterns were examined with indoor and outdoor collections showing distinct variations in three agroecological landscapes: dry mountain, plateau, and semiarid highlands. These agroecological areas are characterized by low and persistent malaria transmission [2]. Further, the association between the host meal source and the infection status of vector species was explored.

## Methods

### Study site

**Fig. 1:**
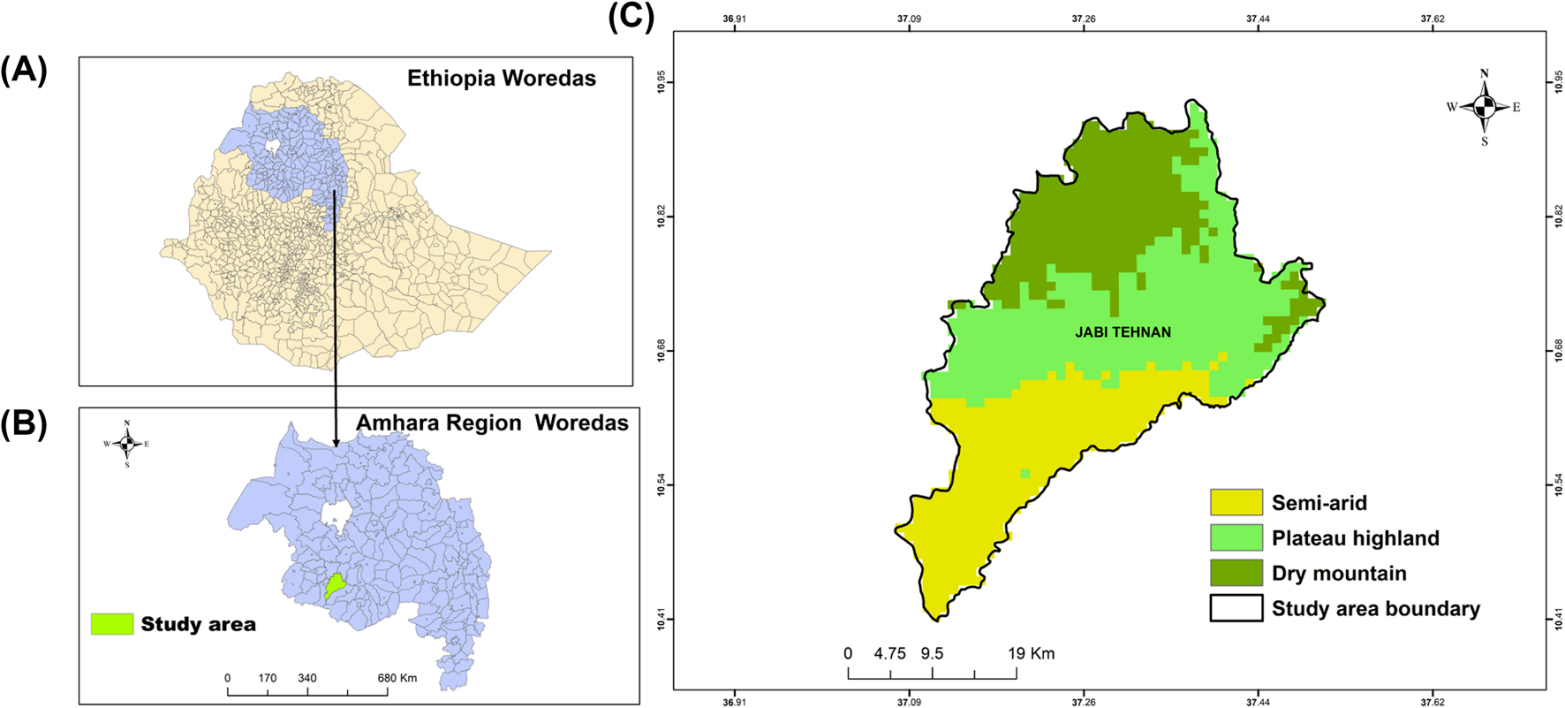
Map of the study area; (**A**) map of Ethiopia, (**B**) Amhara regional administrative division of the study district, and (**C**) study district, Jabi Tehnan

The study was conducted in the Jabi Tehnan district, which is situated in Northwestern Ethiopia’s Amhara Regional State in the West Gojjam Zone (Fig. 1). Malaria is endemic in the district, which consists of 3 small towns and 38 rural villages [27]. An official estimated population is over 228,351 from 45,827 rural and 7927 urban households based on the 179,342-person population report from the 2007 Ethiopian Statistics Service census [28]. The primary economic activity of the inhabitants is small-scale mixed farming, with crop production and animal production being the main sectors. Cattle are the main source of income, followed by horses, sheep, goats, and numerous fowl [27, 29]. The district’s topography is mostly composed of 65% plain land (plateau), 15% mountainous land, 15% undulating land, and 5% valley land. The altitude varies from 1300 m to 2330 m above sea level (asl), with plain (plateau) and dry mountain highlands and subtropical dry areas (semiarid). In addition to having a moderate-to-high mean daily temperature range of 15–20 °C, the region is well known for its heavy rainfall; unimodal distribution of rainfall occurs during the rainy season, which typically spans 4 months, from mid-June to mid-September, with an annual precipitation record of 1356–1720 mm [29]. The area is endemic for malaria, with transmission occurring during the harvest season (autumn), typically lasting from October to December, and during the short rainy season (Spring) between March and May. Houses in the district typically have mud-plastered walls, doors and windows made of furnished wood or metal sheet, and corrugated iron roofs, and most households (about 60%) keep cattle in a separate area from where humans dwell, and in some cases reside in a separate room but shared with humans [27]. Malaria incidence is also sustained throughout the dry months of January and February [30].

### Mosquito field sampling

Engorged mosquitoes collected during a baseline study of malaria transmission in the district were used. Sampling (June 2020 and May 2021) in randomly selected households indoors and outdoors was undertaken using CDC light traps (Model 512, John W. Hock, Gainesville, FL, USA) in different villages in three agroecological zones, namely the dry mountain, plateau, and semiarid highland ecologies found in the district. The selection of representative villages was based on the operational plan of the district’s malaria response team for community engagement. This resulted in clusters within the mountain belt, which we refer to as mountain highland; midland referred to as plateau highland; and lowland labelled as semiarid highland. Traps were deployed indoors and outdoors in selected households each trap day, and each sampling month focused on four randomly selected households in a village, representative of each agroecological setting. A total of eight traps were installed in a chosen village per zone, with one indoor and one outdoor trap placed in each residence. In each sample month, 24 randomly chosen families were targeted from each agroecological region. Targeting several sets of randomly selected houses, the same approach was used to guarantee geographical coverage. The traps were run the next day from 18:00 to 06:00 (local time). After being subdued with chloroform, the caught mosquitoes were sorted and morphologically identified to species level using published keys [31, 32]. Using abdominal observations, the physiological condition of each female was categorized as blood-fed, gravid, half-gravid, or unfed [33]. This study analyzed the engorged cohort only of the anopheline mosquitoes sampled and reported in a recent article by Belay et al. [26].

### PCR detection of vertebrate host meal sources

DNA was extracted from the abdomen of individually engorged anophelines using the Qiagen BioSprint Tissue lysis protocol as described in Ogola et al. [19]. A PCR targeting a 500 bp fragment of the 12S mitochondrial rRNA gene [34] was performed using the methods described by Kamau et al. [35]. Amplicons were verified on 1.5% agarose gel electrophoresis and run against 100 bp DNA HyperLadder (Bioline, Meridian Bioscience, TN, USA). All positive PCR products were purified with the ExoSAP-IT DNA purification kit (USB Corporation, Cleveland, OH, USA) and sent to Macrogen Europe BV (Amsterdam, the Netherlands) for Sanger sequencing using the forward primer. The resulting DNA sequences were cleaned in MEGA v6 [36] and a BLASTn search in the GenBank database was performed for homologous sequences, as previously used by Kamau et al. [35]. Sequences were assigned to species when they had ≥ 98% identity spanning at least 300 bp [35].

### *Plasmodium* infection analysis

We used the extracted abdominal DNA as described by the aforementioned methods by Ogola et al. [19], and DNA pellet was processed for *Plasmodium* screening as outlined in Kinya et al. [37] using non-coding mitochondrial strands (ncMS) primers. This targets the Intergenic spacer (IGS) of the cytochrome-c oxidase subunit 1 (*cox1*) gene for *Plasmodium* fluorescence through a melt reporter Hot Firepol Evagreen fluorescent blend (Solis BioDyne, Tartu, Estonia) with high-resolution melting (HRM) analysis of the mic-QPCR assay [3]. For the assay, 10 µL final reaction volume consisting of 2 µL × 0.5 µM Hot Firepol Evagreen mixed with 1 µL template DNA and primers (ncMS-F-5′-TAGCCGACAAGGAATTTTGC-3′ and ncMS-R-5′CCTTGAATGGAGCACTGGAT-3′) 0.5 µM of each was prepared. Thermal conditions were optimized to initial denaturation at 95 °C for 15 min followed by 40 cycles of polymerizations, each running into denaturation at 95 °C for 20 s, 15 s of annealing at 61 °C, and 20 s of extension at 72 °C, and a final extension at 72 °C for 420 s. Standard reference control from the National Institute for Biological Standards and Control (NIBSC; London, UK) was used as standard reference for the assay. The PCR assay was done with RT-PCR-HRM in a RotorGene Q thermocycler (Qiagen) installed with RotorGene Q software v.2.1 (Qiagen). To further confirm the HRM results, positive samples were run via conventional PCR (with similar reaction mix and cycling conditions) using 2xMyTaqMix (Bioline, Germany), and the output was purified with the ExoSAP-IT DNA purification kit (USB Corporation, Cleveland, OH, USA) and outsourced for sequencing (Macrogen Europe BV, Amsterdam, the Netherlands). All sequence trimming and gene analysis were done as mentioned above for the blood meal DNA using MEGA v6, including a BLASTn search.

### Ethical approval

A review of the study protocol and ethical approval were given by the Regional Public Health Research Ethics Review Committee/RERC/(ref. no.: H/R/T/T/D/5/3) from Amhara Public Health Research Institute, Ethiopia. The research activity was started by providing information about the whole objective of the study to the community and consensus obtained from household owners for the project-related activities done with the directions given by local stakeholders. Informed written consent was obtained from household heads to set up mosquito traps in their homesteads.

### Statistical analyses

The *Plasmodium* infection rates and blood meal indexes (bovine and human blood indices) were expressed as the number of positive specimens of the total number examined for selected species and the proportion compared between indoors and outdoors and between agroecology using a chi-squared test for equality of proportion at 95% confidence intervals (CI). A bipartite graph analysis tool was employed for developing the vector–host association plot [38, 39]. All analysis was implemented in R v. 4.3.2 [40].

## Results

### Distribution of blood-fed specimens by species and trap location

A total of 330 engorged anophelines were processed with sequences successfully assigned to specific host species from 246 specimens. This translated to a success rate of 74.5% (246/330), of which 49.2% were collected indoors and 50.8% outdoors (Table 1). Of the blood-engorged species, *An. gambiae* s.l. was the most common (80.3%), followed by *An. funestus* s.l. (7.3%), and the remaining 12 species were only sparsely encountered (0.3–2.7%) (Table 1). The detections comprised a single host in individual blood meals with no mixed hosts evident. The unsuccessful samples (*n* = 84) either did not amplify (*n* = 23) or sequences had irregularities in chromatograms (*n* = 61), and hence could not be resolved/assigned to any hosts reliably.

**Table 1:** Engorged anophelines analyzed in indoor and outdoor locations in Jabi Tehnan district, Northwestern Ethiopia

*Anopheles species*	No. successfully scored (no. analyzed; success rate)		Total scored (total analyzed, success rate)
	Indoor	Outdoor	
*An. gambiae* s.l	104 (123, 84.6%)	94 (121, 77.7%)	198 (244, 81%)
*An. funestus* s.l	8 (9, 88.9%)	12 (15, 80%)	20 (24, 83%)
*An. coustani s.l*	2 (2, 100%)	7 (7, 100%)	9 (9, 100%)
*An. pretoriensis*	1 (1, 100%)	4 (4, 100%)	5 (5, 100%)
*An. christyi*	1 (3, 33.3%)	4 (5, 80%)	5 (8, 62.5%)
*An. cinereus*	2 (2, 100%)	0 (1, 0%)	2 (3, 66.7%)
*An. demeilloni*	1 (2, 50%)	0 (0, 0%)	1 (2, 250%)
*An. maculipalpis*	0 (1, 0%)	2 (2, 100%)	2 (3,66.7%)
*An. natalensis*	1 (1, 100%)	0 (1, 0%)	1 (2, 50%)
*An. nili*	1 (2, 50%)	0 (0, 0%)	1 (2, 50%)
*An. pharoensis*	0 (0, 0%)	1 (1, 100%)	1 (1, 100%)
*An. squamosus*	0 (0, 0%)	1 (2, 50%)	1 (2, 50%)
*An. marshallii*	0 (0, 0%)	0 (1, 0%)	0 (1, 0%)
*An. rupicolus*	0 (0, 0%)	0 (1, 0%)	0 (1, 0%)
Total	121 (146, 82.9%)	125 (161, 77.6%)	246 (307, 80%)

### Blood feeding indexes

**Fig. 2:**
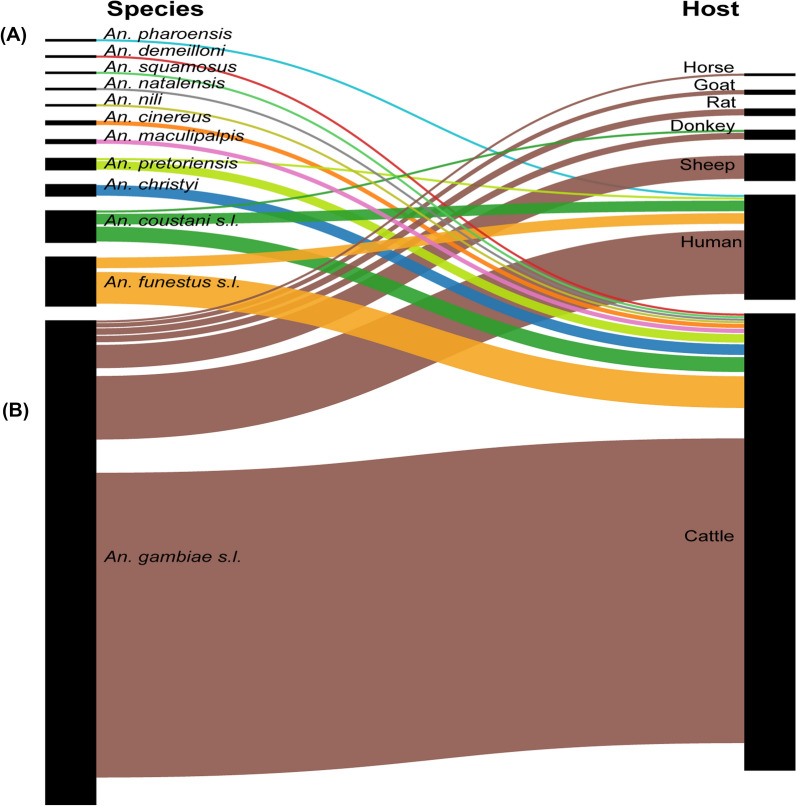
Association between *Anopheles* species with their corresponding vertebrate hosts detected (**A**) upper nodes represent hosts, orange for cattle, purple for humans, green for sheep, yellow for goats, red for donkeys, black for horses, and brown for rats; **B** bottom nodes in light blue color represent mosquito species. Node size represented the total number of mosquito–host interactions, and the length of the node represented the value/sample size for each

The 246 blood-fed mosquitoes successfully profiled were from 12 anopheline mosquito species (Table 1). Of these, cattle served as the most common blood meal source for virtually all the mosquito species accounting for 183/246 of feeds (bovine blood index, BBI = 74.4%) (Table 2; Fig. 2). Only five species (*An. gambiae* s.l., *An. funestus* s.l., *An. coustani* s.l., *An. pretoriensis, and An. pharoensis*) fed on humans as the second most widely utilized host (overall human blood index, HBI: 42/246, 17.1%). Among the species, *An. gambiae* s.l. was the dominant blood-fed mosquito observed, which largely obtained blood from cattle (146/198, BBI = 73.7%), followed by humans (31/198, HBI = 15.7%). This species also exhibited the widest host range (*n* = 7), indicating plasticity in feeding habits. The other species had a narrow feeding range (*n* = 1), having only fed on cattle and/or humans (*n* = 2), *An. funestus* s.l., *An. coustani* s.l., and *An. pretoriensis*. *Anopheles funestus* s.l., the next most abundant blood-fed mosquito, had an estimated HBI and BBI of 25% (5/20) and 75% (15/20), respectively. Overall, the mosquitoes analyzed exhibited a significantly higher zoophagic than anthropophagic tendency for source of blood meals (204/246 versus 42/246; *χ*^2^ = 213.4, *df *= 1,* p* < 0.0001).

**Table 2:** Host feeding profile of *Anopheles* species indoor versus outdoor and estimated blood indices in Jabi Tehnan district, Northwestern Ethiopia

Trap location	*Anopheles* species	Human	Cattle	Donkey	Goat	Horse	Rat	Sheep	Total	HBI (%)	BBI (%)
Indoor	*An. gambiae* s.l	18	76	4	1	0	1	4	104	14.9	62.8
	*An. funestus* s.l	1	7	0	0	0	0	0	8	0.8	5.8
	*An. coustani* s.l	2	0	0	0	0	0	0	2	1.7	0
	*An. christyi*	0	1	0	0	0	0	0	1	0	0.8
	*An. cinereus*	0	2	0	0	0	0	0	2	0	1.7
	*An. demeilloni*	0	1	0	0	0	0	0	1	0	0.8
	*An. natalensis*	0	1	0	0	0	0	0	1	0	0.8
	*An. nili*	0	1	0	0	0	0	0	1	0	0.8
	*An. pretoriensis*	0	1	0	0	0	0	0	1	0	0.8
	Sum	21	90	4	1	0	1	4	121	17.4	74.3
Outdoor	*An. gambiae* s.l	13	70	0	1	1	2	7	94	10.4	56
	*An. funestus* s.l	4	8	0	0	0	0	0	12	3.2	6.4
	*An. coustani* s.l	2	5	0	0	0	0	0	7	1.6	4
	*An. pretoriensis*	1	3	0	0	0	0	0	4	0.8	2.4
	*An. pharoensis*	1	0	0	0	0	0	0	1	0.8	0
	*An. christyi*	0	4	0	0	0	0	0	4	0	3.2
	*An. maculipalpis*	0	2	0	0	0	0	0	2	0	1.6
	*An. squamosus*	0	1	0	0	0	0	0	1	0	0.8
	Sum	21	93	0	1	1	2	7	125	16.8	74.4

### Effect of location on feeding rates

Separating the data by location indoors and outdoors showed similar utilization rates on cattle (90/121 versus 93/125) as a common feeding source for the mosquito species. More species (species richness = 5) fed on humans outdoors than indoors (species richness = 3) and the HBI did not vary between indoor and outdoor (21/121 versus 21/125; *χ*^2^ = 0.01, *df* = 1, *p* = 0.91, Table 2). *Anopheles gambiae* s.l. displayed higher bovine feeding rates that did not differ between indoor and outdoor (76/104 versus 70/94; *χ*^2^ = 0.05, *df* = 1, *p* = 0.82, Table 2). Additionally, HBI for *An. gambiae* s.l. was found not to be different both indoors and outdoors (18/104 versus 13/94; *χ*^2^ = 0.45, *df* = 1, *p* = 0.50, Table 2). More *An. funestus* s.l. mosquitoes fed outdoors on cattle and humans (Table 2).

The feeding relationships and interactions between *Anopheles* mosquitoes and their host species (names described in NCBI open database) characterized found that 74.4% (*n* = 183) had fed on *Bos taurus* species (cattle), 17% (*n* = 42) on *Homo sapiens* (human), and 4.5% (*n* = 11) on *Ovis aries* (sheep). A smaller proportion, approximately 1.8% (*n* = 4), had fed on *Equus asinus* (donkey), 1.2% (*n* = 3) on *Mysateles prehensilis* (rat), 0.8% (*n* = 2) on *Capra hircus* species (goat), and 1 mosquito (0.4%) was detected to have fed on an *Equus caballus* (horse) (Fig. 2).

### Effect of agroecology on blood feeding rates

The abundance of blood-fed *Anopheles* mosquitoes versus their attributed hosts across three adjacent agroecological areas revealed several blood-fed female anopheline were collected in semi-arid (*n* = 130), followed by plateau highland (*n* = 67) and dry mountain highland (*n* = 49) (Table 3). In dry mountain and plateau highlands, the mosquitoes fed at least twofold more indoors (*n* = 40 and *n* = 48) than outdoors (*n* = 9 and *n* = 19), while the opposite was true in semiarid areas: threefold higher outdoors (*n* = 97) than indoors (*n* = 33) (Table 3). Overall, BBI did not vary across the agroecological areas (35/49, 53/67, 95/130 of dry mountain, plateau, and semiarid, respectively; *χ*^2^ = 1.12, *df* = 2, *p* = 0.57). Likewise, HBI: 11/49, 11/67, 20/130 of dry mountain, plateau, and semiarid, respectively, did not vary across the agroecological areas (*χ*^2^ = 1.21, *df* = 2, *p* = 0.55). *Anopheles gambiae* s.l. had the highest feeding host range in semiarid [7] than either dry mountain (*n* = 4) or plateau highland (*n* = 5). The HBI for this species was comparable indoor and outdoor in dry mountain highlands (6/32 versus 1/4; *χ*^2^ = 0.09, *df* = 1, *p* = 0.77), plateau highlands (5/42 versus 1/11; *χ*^2^ = 0.07, *df* = 1, *p* = 0.79), and semiarid ecology (7/30 versus 11/79; *χ*^2^ = 1.40, *df* = 1, *p* = 0.24). Across agroecology, the BBI attributed to this species was relatively higher in the plateau highland (44/53, 83%), followed by dry mountain highland (26/36, 72%) and semiarid (76/109, 69.7%) ecology.

**Table 3:** Host fed upon by different anopheline mosquitoes by agroecology in Jabi Tehnan district, Northwestern Ethiopia

*Anopheles* species	Host	Dry mountain		Plateau highland		Semiarid	
		Indoor	Outdoor	Indoor	Outdoor	Indoor	Outdoor
*An. gambiae* s.l	Cattle	23	3	34	10	19	57
	Human	6	1	5	1	7	11
	Donkey	2	0	1	0	1	0
	Goat	0	0	0	0	1	1
	Horse	0	0	0	0	0	1
	Sheep	0	0	2	0	2	7
	Rat	1	0	0	0	0	2
*An. funestus* s.l	Cattle	4	1	2	1	1	6
	Human	0	3	1	0	0	1
*An. coustani* s.l	Cattle	0	0	0	4	0	1
	Human	1	0	1	2	0	1
*An. pharoensis*	Human	0	0	0	1	0	0
*An. christyi*	Cattle	1	1	0	0	0	3
*An. cinereus*	Cattle	1	0	0	0	1	0
*An. demeilloni*	Cattle	0	0	1	0	0	0
*An. maculipalpis*	Cattle	0	0	0	0	0	2
*An. natalensis*	Cattle	0	0	1	0	0	0
*An. nili*	Cattle	1	0	0	0	0	0
*An. pretoriensis*	Cattle	0	0	0	0	1	3
*An. squamosus*	Cattle	0	0	0	0	0	1
	Total	40	9	48	19	33	97

### *Plasmodium* infection in engorged mosquitoes

We found a low *Plasmodium *spp. infection rate (4.64%) with 13 samples confirmed positive for *P. falciparum* and only 1 with *P. vivax*. Among the mosquitoes positive for *Plasmodium *spp., 79% (*n* = 11) were identified as *An. gambiae* s.l. (*An. arabiensis*), while the remaining three were *An. coustani* s.l., *An. rupicolus*, and *An. funestus* s.l. Most of the positive *An. arabiensis* (*n* = 8) and the *An. coustani* s.l. samples had their blood meal from cattle. Comparatively, the infected *An. funestus* s.l. contained human DNA, while three infected *An. arabiensis,* and *An. rupicolus* were unsuccessfully typed for any host DNA.

## Discussion

This study analyzed the trophic relationships of engorged anophelines surveyed from three agroecological areas in the Jabi Tehnan district of northwestern Ethiopia. The relative abundance of blood-fed mosquitoes was greater indoors in dry mountain and plateau highland areas and outdoors in semiarid areas. In both plateau and dry mountain areas, more human feeding was observed indoors, indicating inherent exposure risk to mosquito bites when asleep at night. However, in these areas, a high degree of zoophagy mainly in cattle was found among indoor sampled mosquitoes. The data indicate the propensity of the mosquitoes to feed outdoors but their preference to rest indoors. This behavior could be influenced by a combination of factors including housing structure, weather variables (temperature), or reduced resting sites (vegetation) [21, 24, 41–43]. *Anopheles* mosquitoes exhibited increased outdoor activity and biting rates in moderate climates, such as semiarid highland regions, compared with cooler highland areas [25]. Conversely, the semiarid environment exhibited higher outdoor blood-feeding rates in animals and humans, indicating a preference for outdoor biting activities and perhaps resting sites. These findings align with previous research in dryland ecosystems [3, 37, 44]. Thus, an interplay between environmental factors, vector behavior, and human activities could drive indoor decisions for blood meals or resting [43].

The feeding pattern was largely driven by *An. gambiae* s.l. mosquitoes, which accounted for > 80% of blood-fed mosquitoes successfully profiled, likely reflecting its relative abundance in the study areas. This species complex is dominated by *An. arabiensis*, which is the most prevalent and primary malaria vector across most ecologies in Ethiopia [2, 13, 14, 45–47]. This is consistent with our findings among the infected samples identified at the species level. The most infected blood-fed specimens were *An. arabiensis*, attesting to its high susceptibility to *Plasmodium* parasites and an efficient malaria vector [45]. We found high bovine feeding in this species consistent with published literature [8, 10, 13, 14, 17], including its plastic behavior of feeding on humans and other hosts. *Anopheles gambiae* s.l. exhibited a high zoophagic tendency with an opportunistic outlook, feeding on various species of vertebrate hosts, including humans, cattle, sheep, donkeys, goats, horses, and rats [8, 19]. Despite humans serving as the second utilized hosts, the HBI for *An. gambiae* s.l. did not show significant differences between the agroecologies nor between indoor and outdoor collections within each agroecology, indicating consistent feeding trends regardless of habitat types [10, 14]. The broad feeding host range for *An. gambiae* s.l. in semiarid areas than the other agroecological zones could be indicative of variation in host community structure.

The ability to feed on humans is an important attribute that has a direct relationship with the vectorial capacity of any vector. Only five species had fed on humans, three of which tested positive for *P. falciparum* (*An. arabiensis*, *An. funestus* s.l., and *An. coustani* s.l.), representing a risk factor for malaria transmission within households and the peri-domestic space. The results buttress their role in malaria transmission in Ethiopia [2, 20, 48] and elsewhere in Africa [3, 11, 14, 16, 37, 44, 49]. It could be considered that the parasite was limited to the midgut given the *P. falciparum* detection in the abdominal blood meals. The presence of cattle DNA in the infected mosquitoes (*An. gambiae* s.l./*An. coustani* s.l.) suggests multiple feeding that included humans, perhaps beyond the detection limits of the current PCR approach employed. The blood feeding analysis likely targets hosts with the most abundant DNA and is limited in detecting or resolving multiple hosts in a single blood meal or previously encountered hosts [50–52]. New methods based on next-generation sequencing approaches have been instrumental in resolving mixed blood meals [51–54], and application could aid insights into host–feeding interactions of these malaria vectors.

*Anopheles funestus* s.l., the second most abundant blood-fed species, demonstrated a more limited host range on cattle and humans. *Plasmodium*-infected *An. funestus* s.l. had DNA from humans, suggesting a close association with humans. Together with its feeding habits being higher in humans outdoors would predict a high likelihood of its importance in residual malaria transmission. Our previous genetic analysis of mosquitoes morphologically scored as *An. funestus* s.l. revealed previously undescribed species that likely represent cryptic vectors [26].

Our results have important implications for malaria transmission risk and control. First, cattle served as a common host source for feeding by the diverse anophelines, including primary and secondary malaria vectors. As alternative hosts, they could reduce the frequency of vectors biting humans, although this has been found to vary by species [55]. However, high attractiveness of malaria mosquitoes to cattle close to human dwellings instead can increase the likelihood of malaria risk to humans [56]. Cattle feeding can sustain malaria vectors via blood meals and enhanced vector survivorship, contributing to malaria persistence. Thus, cattle-targeted interventions or applications in their surrounding areas could selectively affect the survival and fecundity of malaria vectors, thereby contributing to sustainable control. Examples of such interventions include the use of endectocides [46] and insecticide application around cattle enclosures, which should be considered. Second, the difference in resting habits (indoors versus outdoors) among the agroecological settings underscores that targeted approaches must be guided by local epidemiology, especially hinging on vector behavior. If reflected in the abundance trends, then indoor measures such as long-lasting insecticidal nets (LLINs) and indoor residual spraying (IRS) would be more impactful in dry mountain/plateau settings than in semiarid areas.

One limitation of the study is the use of CDC light traps to sample the blood-fed mosquitoes analyzed. This trapping tool predominantly targets host-seeking mosquitoes and could affect interpretation of the blood feeding pattern generated, which could differ depending on the collection method [5, 57]. This method nonetheless captures engorged cohorts as reported in our study and published literature [6]. Additional studies should consider resting collections indoors and outdoors using spray catches and aspiration to maximize the collection of engorged mosquitoes. Another weakness is the detection of *Plasmodium* parasites in the abdomen and not the head/thorax region containing the salivary glands. Thus, our data, based on parasite detection, cannot be used to infer transmission or vectoring capacity. Our detection in the abdomen confirms presence of infection only as found previously (58, 59), but coincidentally in species with established roles as malaria vectors.

## Conclusions

Agroecology influenced catches of engorged mosquitoes indoors versus outdoors although analysis depicted higher overall zoophagic than anthropophagic habits, indicating the importance of alternative hosts other than humans in sustaining malaria vectors. The feeding trend was dictated by *An. arabiensis*, mainly feeding on cattle while being opportunistic and feeding also on a broad range of hosts including humans and other peri-domestic hosts. Our results demonstrate communal feeding on cattle by diverse anophelines, including primary and secondary malaria vectors, underscoring the importance of cattle-targeted interventions for consideration in the toolbox to sustainably control malaria in the study areas. The identification of cow DNA in *Plasmodium*-positive mosquitoes stresses the need for further research to elucidate the role of livestock in malaria transmission cycles besides targeting cattle in malaria control. By understanding the interactions between mosquitoes, hosts, and the environment, we can develop more targeted and effective interventions to reduce malaria burden in endemic areas and ultimately work toward the goal of malaria elimination.

## Data Availability

The datasets used and/or analyzed during the current study are included in this manuscript.
